# The complete mitochondrial genome of the mealy plum aphid, *Hyalopterus pruni* (Hemiptera: Aphididae)

**DOI:** 10.1080/23802359.2020.1832934

**Published:** 2020-11-03

**Authors:** Yanxin Liang, Zhenyong Du, Fan Song, Jia He

**Affiliations:** a Department of Entomology and MOA Key Lab of Pest Monitoring and Green Management College of Pant Protection, China Agricultural University, Beijing, China; b Institute of Plant Protection, Academy of Ningxia Agriculture and Forestry Science, Yinchuan, China; c Ningxia Key Laboratory of Plant Disease and Pest Control, Yinchuan, China

**Keywords:** Mitochondrial genome, Aphididae, *Hyalopterus pruni*

## Abstract

This study completes the sequencing and annotation of the mitochondrial genome (mitogenome) of *Hyalopterus pruni* (Hemiptera: Aphididae) by using the high-throughput sequencing. The mitogenome is a typical circular DNA of 15,410 bp with 86.2% A + T content, and consists of 13 protein-coding genes, 22 tRNA genes, 2 rRNA genes, a repeat region between *tRNA-Glu* and *tRNA-Phe*, and a control region. The gene order follows the putative ancestral arrangements of insect mitogenome. All 13 protein-coding genes start with codon ATN and terminate with TAA or a single T. All tRNA genes have typical clover-leaf structure except for *tRNA-Se*r^AGN^. The control region is 638 bp in length with 86.0% A + T content. The phylogenetic tree supports the monophyly of Aphidini and Macrosiphini in Aphidinae and the sister relationship between *Hyalopterus pruni* and *Schizaphis graminum*.

Aphids are one of the most destructive agricultural pests with complicated heteroecious life circles, elaborate pleomorphism, diverse host plants, and sophisticated symbiotic association (Wang et al. 2015; Xu et al. 2020). The mealy plum aphid, *Hyalopterus pruni* (Geoffroy) is an important pest in orchards, which seriously damages peach, plum and apricot around the world (Lozier et al. 2007). This study firstly presented the complete mitogenome of *H. pruni* from subfamily Aphidinae, which was obtained by high-throughput sequencing. The aphid samples were collected from Balanghu farm, Wuzhong, Ningxia Province (38°32′45″N, 106°22′38″E). The voucher specimen (NO. VAph-0128) was deposited in the Entomological Museum of China Agricultural University (CAU), Beijing, China. The complete mitogenome of *H. pruni* was obtained by high-throughput sequencing on Illumina NovaSeq 6000 platform (San Diego, USA). The mitogenome was assembled by IDBA-UD 1.1 (Peng et al. 2012), and the preliminary annotation was performed on MITOS (Bernt et al. 2013) to delimit gene boundaries. The secondary structure of tRNA genes were predicted by tRNAscan-SE Search Server v.2.0 (Schattner et al. 2005). Protein-coding genes and rRNA genes were identified by alignment with homologous genes of published aphid mitogenomes using Geneious 10.1.3 (http://www.geneious.com/).

The complete mitogenome of *H. pruni* is 15,410 bp in length containing 13 protein-coding genes (PCGs), 22 tRNA genes, 2 rRNA genes, a repeat region, and a control region (Boore 1999). Twenty-three genes were transcribed on the majority strand (J-strand), the other fourteen genes were coded on the minority strand (N-strand). The gene order follows the putative ancestral arrangement of insects (Clary and Wolstenholme 1985; Cameron 2014). In *H. pruni*, 10 overlaps (1 ∼ 20 bp) between adjacent genes were found, the longest overlap is 20 bp between *ATP8* and *ATP6*. The intergenic spacers were detected in 13 locations, ranging from 1 ∼ 16 bp.

The nucleotide composition of *H. pruni* is 41.2% A, 45% T, 8.6% C, 5.1% G, with significant A + T biased (86.2%). It is slightly T skewed (AT-skew= −0.04) and strongly C skewed (GC-skew= −0.26). All PCGs use the typical start codon ATN, five with ATA, five with ATT, two with ATG, and the remainders with ATC. There are 11 PCGs terminated with the most common stop codon TAA, whereas *COI*, *ND4* uses incomplete termination codons with single T, which is commonly occurred in Hemiptera insects (Li et al. 2016; Li et al. 2017).

The 22 tRNA genes are from 62 to 73 bp in length. Most of the tRNA can be folded into the typical clover-leaf structures except for *tRNA-Se*r^AGN^ in which the DHU arm forms a loop instead of a typical DHU arm. The length of rrnL and rrnS are 1260 bp and 771 bp, respectively. The rrnL gene and rrnS gene have an A + T content of 85.3% and 84.3%, respectively. The control region was rich in A + T (86.0%) and 638 bp in length. There is a repeat region between *tRNA-Glu* and *tRNA-Phe* in *H. pruni* with 2 tandem repeats, which is an interesting feature and reported in aphid mitogenomes several times (Wang et al. 2014; Wang et al. 2015).

Phylogenetic analysis was generated by the maximum-likelihood (ML) method based on 16 aphid mitogenome sequences (Figure 1). The result supports the monophyly of Hormaphidinae, Eriosomatinae, Greenideinae, and Aphidinae. Meanwhile, the monophyly of Aphidini and Macrosiphini in Aphidinae are well recovered with high support values. The sister relationship between *H. pruni* and *Schizaphis graminum* is also highly supported.

**Figure 1.**
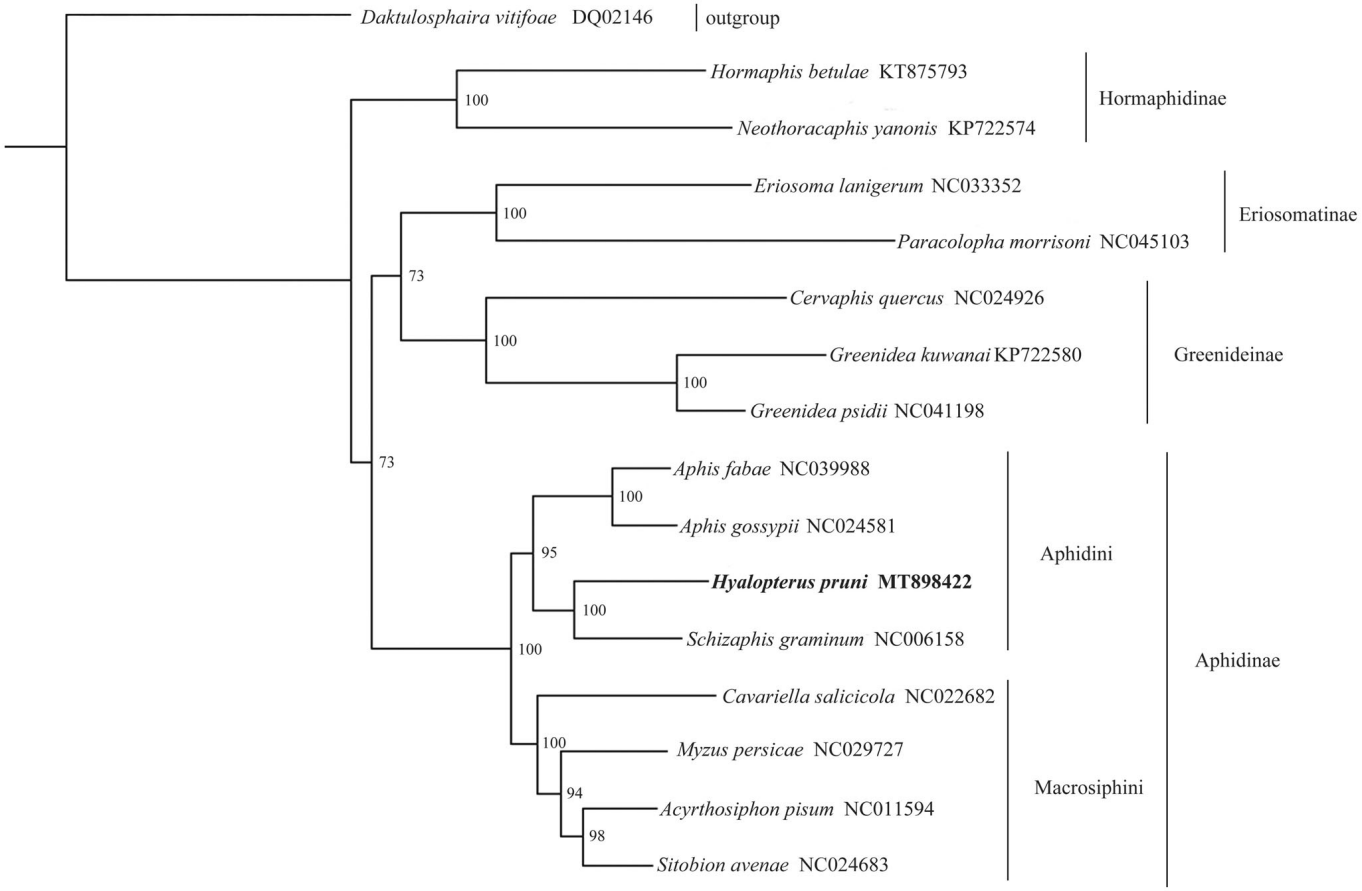
Phylogenetic relationship was inferred based on 13 PCGs of 16 aphid mitogenomes, with GenBank accession numbers also shown. The phylogenetic tree was generated from IQ-TREE 1.6.5 (Trifinopoulos et al. 2016) under the GTR + I + G model. The node supports are the bootstrap values obtained with 1000 replicates.

## Data Availability

The mitogenome and raw sequencing data in this study are available in GenBank (https://www.ncbi.nlm.nih.gov/) under the accession numbers of MT898422 and PRJNA663207.
